# Molecular Identification and Phylogenetic Analysis of *Trypanosoma evansi* with Assessment of Associated Risk Factors in Camels (*Camelus dromedarius*) Across Ten Districts of Punjab, Pakistan

**DOI:** 10.3390/vetsci12111055

**Published:** 2025-11-02

**Authors:** Mian Abdul Hafeez, Faiza Aslam, Muhammad Saqib, Muhammad Hammad Hussain, Muntazir Mehdi, Ali Hassan, Adeel Sattar, Atique Ahmed Behan

**Affiliations:** 1 Department of Parasitology, University of Veterinary and Animal Ssciences, Lahore 54000, Pakistan; abdul.hafeez@uvas.edu.pk (M.A.H.); muntazir.mehdi@kuleuven.be (M.M.); 2 Department of Livestock & Dairy Development, Lahore 54000, Pakistan; faizadr@yahoo.com; 3 Department of Clinical Medicine and Surgery, University of Agriculture, Faisalabad 38000, Pakistan; drsaqibm@uaf.edu.pk (M.S.); dr.alihassan@uaf.edu.pk (A.H.); 4 Department of Animal & Veterinary Sciences, College of Agricultural & Marine Sciences, Sultan Qaboos University, Al Khoudh, Muscat 123, Oman; a.behan@squ.edu.om; 5 Department of Pharmacology and Toxicology, University of Veterinary and Animal Sciences, Lahore 54000, Pakistan; adeel.sattar@uvas.edu.pk

**Keywords:** camel, *T. evansi*, surra, phylogenetic analysis, risk factors, serum biochemistry, Pakistan

## Abstract

**Simple Summary:**

This study investigated the molecular detection, phylogenetic analysis, and risk factors associated with *Trypanosoma evansi* infection in camels from ten districts in Punjab, Pakistan. Blood samples from 400 camels were analyzed using microscopic examination and PCR assays. The study found a higher prevalence of *T. evansi* through PCR (14.8%) compared to microscopy (8.3%). Phylogenetic analysis showed 100% homology with isolates from India, Sudan, Malaysia, Egypt, and Kenya. The study identified female gender and being in Southern Punjab as significant risk factors for *T. evansi* infection. The research provides new molecular and phylogenetic data on *T. evansi* isolates from the study area.

**Abstract:**

Trypanosomiasis significantly impacts camel health and productivity, posing a major challenge to food security in regions with large camel populations. In this study, we investigated the microscopic and molecular prevalence, performed phylogenetic analysis, and explored risk factors associated with *Trypanosoma evansi* (*T. evansi*) infection in 400 randomly selected suspected camels (*Camelus dromedarius*) from 10 districts of Punjab, Pakistan. Blood samples were collected for microscopic examination of Giemsa/Field’s-stained smears, and three PCR primer sets (ITS1CF/BR, pMUTec, RoTat 1.2) were used to detect the presence of *T. evansi*. PCR-based prevalence was higher (14.8%; CI 11.4–18.6) as compared to the microscopic examination (8.3%; CI 5.7–11.4) of samples. The targeted primers amplified DNA fragments of 210, 205, and 478 base pairs, respectively. Phylogenetic analysis showed 100% homology between local isolates and those from India, Sudan, Malaysia, Egypt, and Kenya. Risk analysis identified female gender (OR 2.1) and being in Southern Punjab (OR: 1.9) as significant factors associated with disease. Significantly (*p* < 0.05) reduced total protein (5.51 ± 0.05), albumin (2.77 ± 0.04), and globulin (2.57 ± 0.06) levels were found in PCR-positive camels. This study provides new molecular and phylogenetic data on *T. evansi* in Pakistan.

## 1. Introduction

Among the numerous domesticated livestock species, the camel is an important multipurpose animal used for milk, meat, wool and carriage in arid and semi-arid zones of the world. Camels play a significant role in the endurance of the poor people [1]. Camels are well adapted to desert regions, capable of surviving long periods without water and tolerating wide fluctuations in body temperature. Pakistan ranks eighth in the world amongst camel-rearing nations with 1.1 million heads [2]. Pakistani camel breeds are among the best milk-producing breeds in the world [1]. Camel milk plays a significant role in the food chain and disease control strategies due to its exceptional nutritional and therapeutic properties [3].

Camels suffer from various parasitic diseases that affect their health and production. Trypanosomiasis, initially identified in India and referred to as Surra, has now become one of the most significant hemoprotozoan diseases affecting camels worldwide, including in Pakistan [4]. The genus trypanosoma has multiple pathogenic protozoan species like *Trypanosoma evansi* (*T. evansi*), *Trypanosoma Brucei* (*T. brucei*), *Trypanosoma vivax* (*T. vivax*), *Trypanosoma equiperdum* (*T. equiperdum*) and *Trypanosoma congolense* (*T. congolense*). All these species have the potential to infect a wide range of domesticated animals, including those of camelid, equine, caprine, ovine, and canine origin [5]. One of the most important hemoparasites is *Trypanosoma evansi,* affecting the domestic livestock in many countries, including South and Central America, Africa, and Asia. *Trypanosoma evansi* is recognized as a non-cyclic parasitic disease predominantly transmitted through mechanical means, having evolved from T. brucei to extend its range beyond the traditional tsetse belt [6,7]. Mechanical transmission is facilitated by biting flies belonging to genera such as Tabanus and Stomoxys [4], wherein the trypanosome persists briefly in the vector’s oral cavity without undergoing developmental stages [6,8,9,10]. Additional transmission pathways include oral exposure among carnivores consuming fresh infected meat, while in South America, the vampire bat (*Desmodus rotundus*) serves both as a vector and reservoir, transmitting the parasite [7,11].

Trypanosomiasis is clinically manifested by anorexia, weight loss, poor body condition, subcutaneous edema, atrophied thigh muscle, pale mucous membrane of conjunctiva, lacrimation, icterus, swelling in testis, and nervous signs. The condition can manifest in either an acute or chronic form, potentially persisting for several months or even years [7]. The clinical manifestations of the disease in camels vary depending on its progression. In the acute phase, affected camels commonly experience fever, edema, lethargy, and progressive weight loss. As the disease becomes chronic, symptoms may include anemia, immunosuppression, infertility, abortions, and a noticeable reduction in milk yield. Advanced cases can present with neurological signs such as ataxia, paralysis, and incoordination [11,12]. The rainy season in Pakistan is considered the Surra season, wherein the number of biting flies increases [13].

*Trypanosoma evansi* is recognized as a pathogen of significant medical and economic consequence, affecting cattle, buffaloes, pigs, and companion animals in regions of Asia and Africa. Infection in Asian cattle is characterized by fever, weight loss, neurological symptoms, and abortion. In African cattle, it is considered as mild or negligible infection [7]. In equines, clinical presentation frequently includes acute and profound anemia. The condition, commonly known locally as ‘mal de cadeiras’ in the Brazilian Pantanal, is associated with marked emaciation, immunosuppression, and severe neurological disturbances, and it can be fatal if untreated [14]. In canines and experimental rabbits, infection leads to pyrexia, lymphadenopathy, anemia, and considerable biochemical abnormalities [15,16]. To date, three cases of human infection with fever of unknown origin have been reported in India and Vietnam [6,8].

The monetary effects of this protozoa are often underestimated and include morbidity rates of up to 30%, mortality rates of approximately 3%, and abortion. Overall, the disease is characterized by high morbidity and mortality rates, particularly in herds that have not previously been exposed to the pathogen [17]. Diagnosis of the disease through conventional parasitological diagnostic techniques is better in the acute stage of disease. The chronic stage of trypanosomiasis is distinguished by low levels of parasitemia, making a reliable parasitological diagnosis challenging [18]. Polymerase chain reaction (PCR) is performed for diagnosis and identification of the specific protozoan species. This technique is helpful in confirmation of diseased animals, as well as identification of carrier stage. Polymerase chain reaction (PCR) is a highly specific and sensitive diagnostic tool commonly employed for the confirmation of blood parasites [19].

Although various studies investigating *T. evansi* prevalence have been conducted in different camel-populated areas of Pakistan [9,20,21,22,23,24], precise and concise information about the infection over a wider geographical area is scarce. This study aimed to investigate the presence of the disease using a sensitive and efficient polymerase chain reaction (PCR) technique and to examine the genetic relationships between local isolates and existing species available in GenBank, including their DNA sequences. Furthermore, this study assessed the sero-biochemical parameters of camels and examined the association of various hypothesized risk factors influencing the spread of camel trypanosomiasis in the targeted regions.

## 2. Materials and Methods

### 2.1. Study Area and Sample Collection

**Figure 1:**
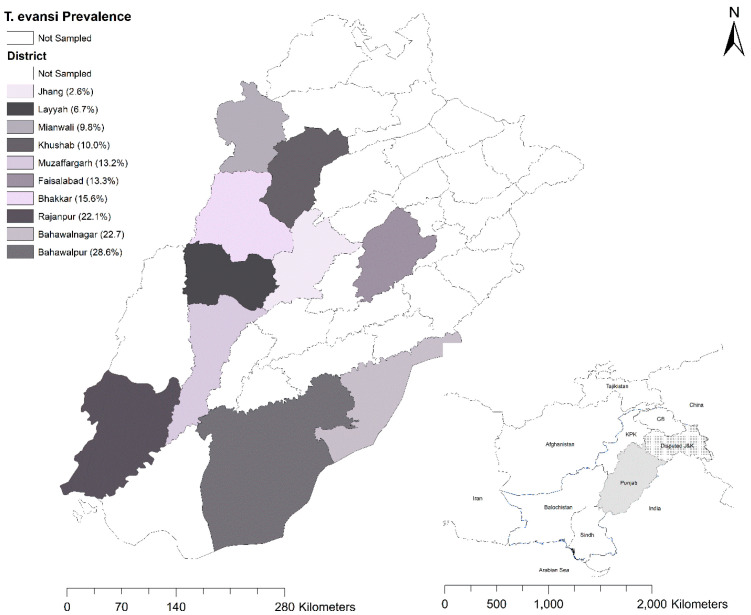
Map of Punjab displaying sampled districts and PCR-based prevalence in 400 camels.

The study was conducted in the Punjab province of Pakistan. The ten districts included in this study were from the Northern (Mianwali and Khushab), Central (Bhakkar, Faisalabad, and Jhang), and Southern Punjab (Bahawalnagar, Bahawalpur, Layyah, Muzaffargarh, and Rajanpur) provincial zones (Figure 1). The estimated camel population of the study area was 96,966 camels [25].

The minimum required sample size (n = 385) was calculated for a disease with 50% expected prevalence, a 95% confidence level, and 5% desired absolute precision [26]. During this study, blood samples (n = 400) were randomly collected from camels of various ages and sexes across 10 districts of Punjab, Pakistan. The random sampling procedure was used to seek a representative inclusion of camels across all zones, with each zone contributing proportionally to the required sample size. Between April 2020 and December 2021, camel holdings belonging to consenting owners in each district were visited. Within each holding, all camels underwent clinical examination, after which those identified as clinically suspected were assigned numbers for random selection. The lottery method was then employed to ensure unbiased selection.

Samples were collected from camels suspected of hemoparasitic infection based on clinical history and signs (fever, wight loss, lethargy, emaciation, etc.) regardless of breed, age, or sex. Approximately 10 mL blood was drained from the jugular vein by a sterile syringe. Camel blood was transferred into a sterile K3. EDTA (Ethylene diamine tetra acetic acid) tube (Atlas-Medo-O-Vae Francisco, Wuhan, China) for microscopy and PCR, while 7 mL was transferred into a plain vacutainer (Biovac^TM^, Cape Town, South Africa) for serum separation [27]. Two apparently healthy and PCR-negative camels from each district were included as controls. Preserved blood samples were transported to the Molecular Parasitology Laboratory (UVAS, Lahore) in ice boxes. Data on all relevant variables, including age, sex, locality, physical appearance, tick infestation, previous illness, deworming status, herd size, and management, were collected using a pre-designed survey form during the sampling process for the investigation and assessment of risk factors associated with *T. evansi* infection in the studied camels.

### 2.2. Microscopic Examination

**Figure 2:**
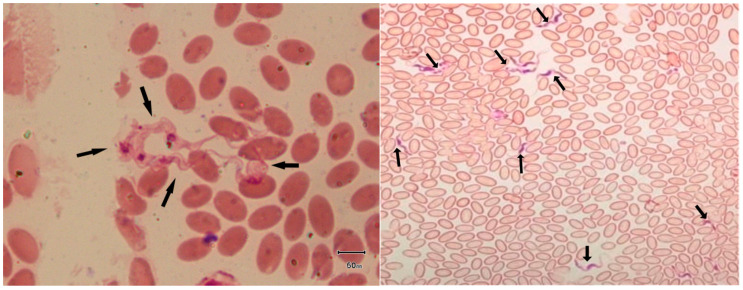
Field/Giemsa staining revealed trypanosomes in the extracellular space of red blood cells of camels under an oil immersion lens (100×) of a compound microscope (Black arrows).

Thin and thick blood smears were processed after fixation and stained by Giemsa/Field stain. Primary screening of blood smears was conducted by using light microscopy at 40× and 100× according to the method described by Hoare to identify trypanosomes (Figure 2) [28]. Blood samples with trypanosomes were recorded as positive and preserved at −20 °C until further analysis.

### 2.3. DNA Extraction and PCR Amplification

All collected blood samples (n = 400) were used for DNA extraction using the WizPrep™ gDNA Mini kit (Wizbiosolutions Inc., Seongnam-si, Republic of Korea) following the manufacturer’s instructions. Purity and concentrations of DNA extracted from the samples were calculated using nano drop and gel electrophoresis techniques, as described previously [29]. For DNA amplification and molecular detection of trypanosomes, PCR was performed using three sets of primers targeting variable genes (Table 1).

The primers specified in the above table were employed to amplify DNA fragments under the following PCR cycling conditions. For ITS1CF/BR primers [30], the reaction began with an initial denaturation step at 95 °C for 3 min, followed by 35 cycles of denaturation at 95 °C for 30 s, annealing at 58 °C for 30 s, extension at 72 °C for 30 s, and a final extension at 72 °C for 7 min. For the pMUTec primers [31], the cycling protocol included an initial denaturation at 94 °C for 3 min, followed by 35 cycles of denaturation at 94 °C for 30 s, annealing at 60 °C for 30 s, extension at 72 °C for 30 s, and a final extension at 72 °C for 5 min. Similarly, for RoTat 1.2 primers [32], the PCR conditions involved an initial denaturation step at 94 °C for 3 min, 35 cycles of denaturation at 94 °C for 30 s, annealing at 58 °C for 30 s, extension at 72 °C for 30 s, and a final extension at 72 °C for 7 min.

The PCRs were carried out in a 25 µL total volume consisting of 12.5 µL of PCR Master Mix (including Taq DNA polymerase, dNTPs, MgCl_2_, and buffer), 1 µL of each primer (10 µM), 2 µL of DNA template (50–100 ng/µL), and nuclease-free water to reach the final volume. Positive controls included DNA extracted from confirmed Trypanosoma-positive samples, while negative controls consisted of nuclease-free water in place of the DNA template. Both positive and negative controls were incorporated in every reaction to ensure accuracy and avoid contamination.

### 2.4. Gel Electrophoresis

**Figure 3:**
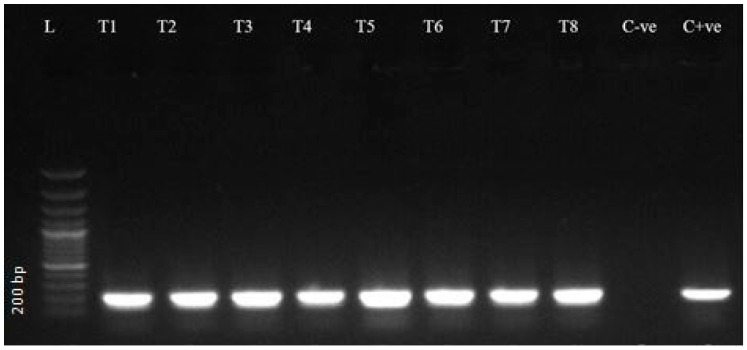
Agarose gel with PCR amplification of RoTat 1.2 (205 bp) of *T. evansi* from camels. Lane L: 100 bp molecular weight marker (Ladder, Thermo Fisher), C + ve (control positive), and C-ve (control negative) with current study positive isolates Lane: T1–T8.

**Figure 4:**
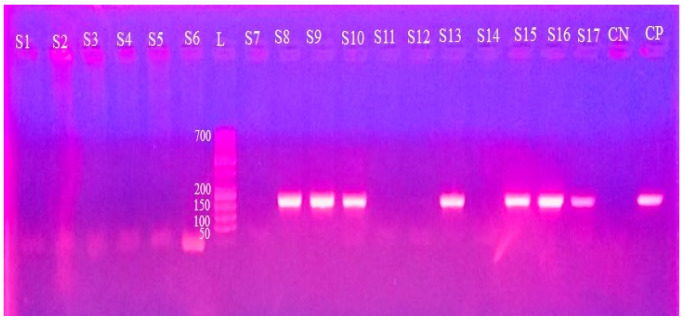
Agarose gel (1.5%) electrophoretogram with ethidium bromide staining displaying pUMtec PCR product of 210 bp from *T. evansi* isolates (different camel samples). L, 50-base pair ladder; CP, control positive; and CN, control negative.

**Figure 5:**
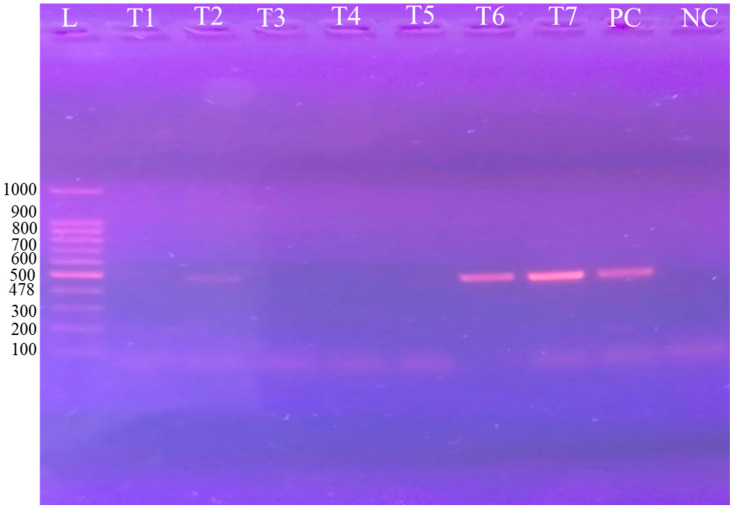
Agarose gel (1.5%) electrophoretogram ethidium bromide staining showing ITS-I CF/BR PCR products of 478 bp derived from *T. evansi* isolates from camel blood samples. L, 100-base pair marker; PC, positive control; and NC, negative control.

Agarose gel (1.5%) was processed and stained with ethidium bromide to evaluate the amplicons generated by PCR. Electrophoresis was executed at 113 volts and 230 mA for 35 min to visualize the amplified product in gel documentation system (Bio Rad Laboratories, Hercules, CA, USA). A DNA ladder of 100 bp (Thermo Scientific^TM^, Waltham, MA, USA) was run along with the PCR amplicon as a molecular weight marker (Figure 3, Figure 4 and Figure 5, Supplementary Figures S1–S3) [33].

### 2.5. Sequencing and Phylogenetic Analysis

The presence of *T. evansi* was confirmed by sequencing. Twelve randomly selected PCR-positive products were purified by using the GeneJET Gel Extraction and DNA Cleanup Kit Thermo Scientific^TM^ according to the manufacturer’s instructions ((Thermo Fisher Scientific™, Waltham, MA, USA). Sequencing was performed unidirectionally using the Sanger method, where DNA was read from a single end. The sequence analysis of the respective target regions using ITS1CF/BR, pMUTec (F/R), and RoTat1.2 (F/R) primers was performed. The obtained oligonucleotide sequences were entered into NCBI (National Center for Biotechnology Information) in BLAST+ version 2.15.0 (Basic Local Alignment Search Tool) to match with existing sequences in GenBank. All deposited sequences were assigned accession numbers (Table 2). Allied sequences were retrieved from GenBank after carrying out a BLAST search, aligned via MUSCLE to create a phylogenetic tree using the maximum likelihood technique by applying Neighbor-Join.

### 2.6. Serum Biochemistry

Serum was separated from the collected samples and stored at −20 °C until further analysis. Biochemical analyses were performed using a clinical chemistry analyzer (Metrolab 1600 DR, Wiener Lab, Rosario, Argentina), following the manufacturer’s instructions. The parameters assessed included serum total protein (g/L), albumin (g/L), A/G ratio, and glucose (g/L). The serum total protein concentration was determined using the biuret method, and albumin was quantified using the bromocresol green dye-binding method. The A/G ratio was calculated by dividing the albumin concentration by the globulin concentration (globulin = total protein − albumin). Glucose levels were measured using the glucose oxidase–peroxidase (GOD-POD) method. The values obtained for infected camels were compared to those of PCR-negative camels.

### 2.7. Statistical Analysis

Statistical analyses were conducted using the R programming language (version 4.5.1). Prevalence percentages, along with their corresponding 95% binomial exact confidence intervals (CI), were calculated. Univariate analysis was conducted to determine variables associated with prevalence. Categorical variables were analyzed using both the Chi-square test and Fisher’s exact test, and odds ratios with confidence intervals were calculated. All variables yielding *p*-values less than equal to 0.25 were included in the binary logistic regression analysis. A backward stepwise approach was subsequently employed to remove confounders (*p* > 0.05), thereby retaining only variables showing significant associations in the final model [34]. Model fit was assessed using the Hosmer–Lemeshow test and Nagelkerke R-square values. Agreement between the two diagnostic tests (microscopy and PCR) was evaluated using Cohen’s Kappa statistic. Comparisons of serum biochemical parameters were made using *t*-tests. Map construction was carried out with ArcGIS software (Version 10.4; ESRI, Redlands, CA, USA).

## 3. Results

### 3.1. Microscopic Findings

Microscopy of stained blood smears revealed Trypanosoma characterized by a long, slender free flagellum in the extracellular space (Figure 2). Microscopic examination identified 33 positive samples out of 400, resulting in an overall prevalence of 8.3% (CI 5.7–11.4). The prevalence varied across districts, with the highest rates observed in Bahawalpur (14.3%) and Rajanpur (14%), followed by other districts with progressively lower rates. Fisher’s exact test (FET) indicated the prevalence differences between districts was not significant; *p* = 0.293 (Table 3).

### 3.2. Molecular Detection Through PCR

PCR revealed 59 (14.8%, CI 11.4–18.6) positive samples (Table 3). The prevalence recorded in different districts was not significantly different (FET, *p* = 0.097). Consistent with the microscopic findings, Bahawalpur exhibited the highest value (28.6%, CI 8.4–58.1), while Jhang reported the lowest (2.6%, CI 0.1–13.5), where no positive samples were detected by microscopy.

### 3.3. Comparison of the Diagnostic Performance

Out of 400 samples, 33 were positive and 367 were negative on microscopy, whereas 59 positives and 341 negatives were recorded by PCR. The comparison of microscopy against PCR (assumed as the gold standard) revealed a low positive predictive value (24.2%), as many microscopy positives were PCR-negative. The negative predictive value was better at 86.1%, making microscopy more reliable for ruling out *T. evansi* than confirming it. The observed agreement was 81.0%; however, the Kappa statistic of 0.076 showed only slight agreement between the two tests, with *p* = 0.108 (Table 4 and Table 5).

### 3.4. Phylogenetic Analysis

**Figure 6:**
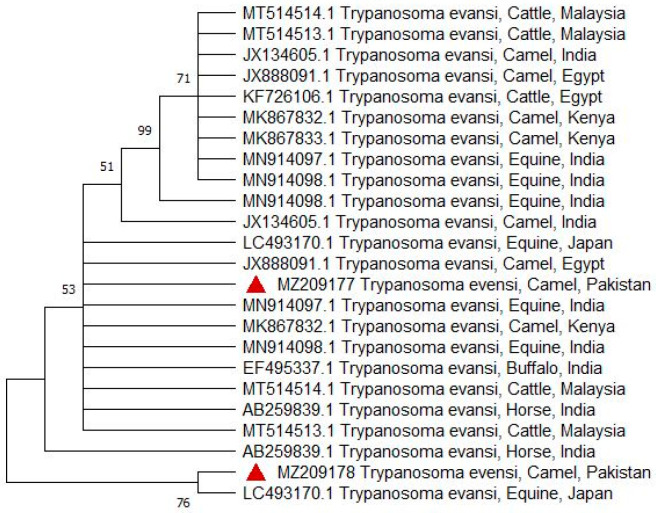
Phylogenetic associations between the samples in the current study and previously published reference sequences were determined based on the RoTat 1.2 (variable surface glycoprotein) gene. These sequences were retrieved from GenBank. A phylogenetic tree was constructed, with clades indicating the percentage similarity at a bootstrap value of 1000. The isolates from the current study are marked with red triangles, while the codes are the accession numbers obtained from NCBI. The main horizontal line indicates the 71% similarity to their ancestor isolated from MN914098.1 Trypanosoma evansi, equine, India. The second branch indicates 99% similarity to their ancestor, JX134605.1 Trypanosoma camel, India. The third branch indicates 51% similarity to their ancestor *Trypanosoma evansi* isolated from different countries, i.e., Japan, Egypt, Malaysia, and India, respectively. The fourth branch indicates 53% similarity to the isolate with AB259839.1 *Trypanosoma evansi*, Horse India. All the branches are linked with their common ancestor via the main root and show 76% similarity to their common ancestor.

**Figure 7:**
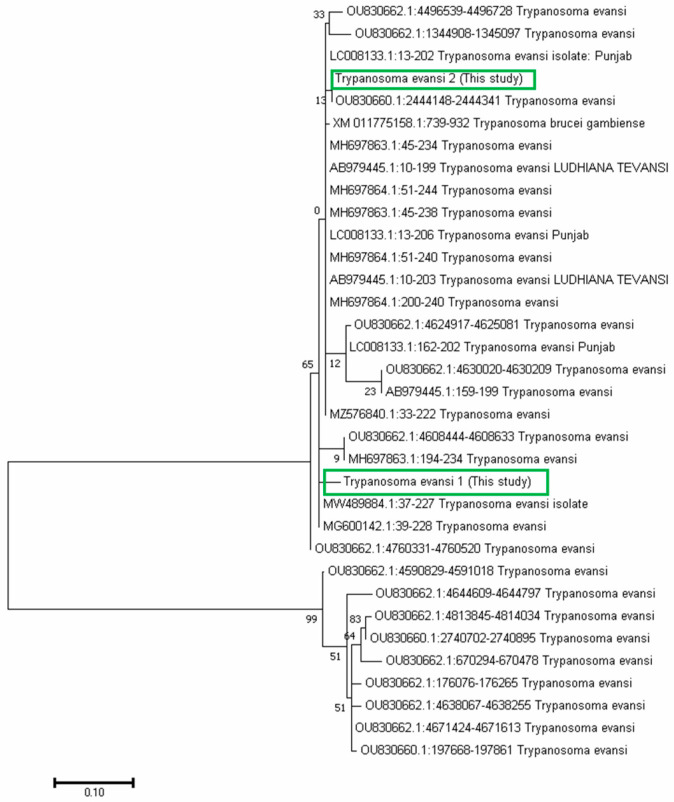
pMUtec (repetitive sequence of 237 bp)-based Phylogenetic relationships between samples in the present investigation and other reference sequences recovered from GenBank. Clade indicates percentage similarities at a bootstrap value of 1000. The codes are the accession numbers obtained from NCBI. The current study isolates, isolate *Trypanosoma evansi* 1 and isolate 2, show 99 percent similarity to their common ancestor, indicated by the main branch root.

**Figure 8:**
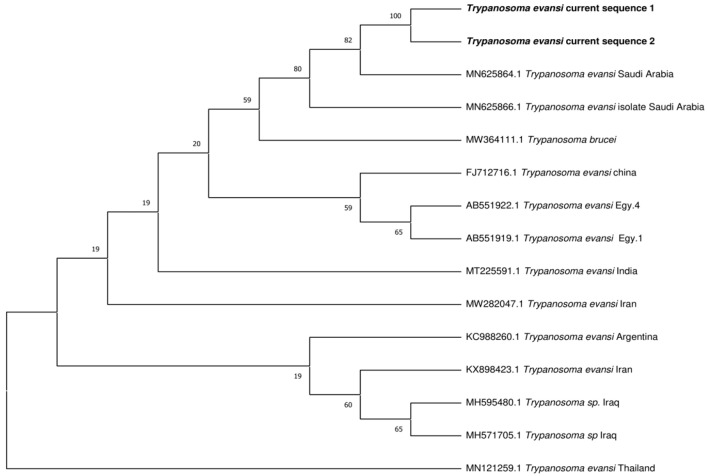
Phylogenetic relationship of Pakistan isolates of *Trypanosoma evansi* with other trypanosomes parasites based on the (ITS-I) gene.

Sequencing and phylogenetic analysis, based on Rotat 1.2VSG (205 bp), pUMtec (210 bp) and ITS1CF/BR (478 bp), confirmed the presence of *T. evansi* in 6 out of 12 PCR products (Figure 6, Figure 7 and Figure 8).

### 3.5. Risk Factors Associated with T. evansi Prevalence

To identify significant variables for performing a binary logistic regression, a univariable analysis was performed on 12 variables (Table 6). District data was further categorized into zones depending on the location. Age (three groups) and herd size (four groups) were also categorized into different groups. The analysis indicated that prevalence of *T. evansi* was associated with gender. Camels from southern Punjab (19.4%, CI 13.8–23.7) districts were found more likely (OR 1.9, CI 1.10–3.38) to test positive. Female camels had higher PCR-based prevalence (18.3%, CI 13.7–23.7) and their odds for testing positive were significantly (*p* = 0.01) higher as compared to males (OR 2.4, CI 1.22–4.51). The prevalence was significantly (*p* < 0.01) high in tick infested camels (20.7%, CI 15.4–26.8) and they were 2.9 times more likely to test positive. Camels kept under desert housing conditions with sandy floors had significantly (*p* = 0.003) higher prevalence of *T. evansi* (19.6%, CI 14.5–25.6), and were found 2.4 times more likely to test positive. Although, numerical differences were observed but the analysis showed no significant (*p* > 0.05) association between *T. evansi* prevalence and age groups, physical appearance, presence of other livestock, fly control, feeding and watering practice, purpose and herd sizes (Table 6). Prior to moving forward with multivariable analysis collinearity was checked. No evidence of multicollinearity was recorded among predictors.

All variables with *p* < 0.25 were included in the initial binary logistic regression model and a backward stepwise exclusion method was used to remove non-significant (*p* > 0.05) variables until only significant (*p* < 0.05) variables were retained. The final model indicated that camels from Southern Punjab (OR 1.9, CI 1.05–3.35), female camels (OR 2.2, CI 1.11–4.24), those with tick infestation (OR 2.6, CI 1.37–4.79), and those kept in sand-based housing (OR 2.2, CI 1.16–3.99) were more likely to test positive for *T. evansi* in the sampled population (Table 7). The model explained 12.8% of the variation (Nagelkerke R^2^ = 0.128), and the Hosmer–Lemeshow test (χ^2^ = 7.038, *p* = 0.533) showed a good fit between predicted and observed outcomes.

### 3.6. Serum Biochemical Findings

An Independent *t*-test revealed significant differences in serum biochemical parameters between *T. evansi*-infected camels (n = 59) and healthy controls (n = 339). PCR-positive camels had significantly (*p* < 0.01) lower levels of total protein and albumin and globulin values when compared with those who were found negative. These findings indicated that *T. evansi* infection significantly altered the serum protein profile in positive camels (Table 8).

## 4. Discussion

The camel contributes imperatively to the socioeconomic elevation of a state, serving as both a draught animal and as a protein source. Protozoan diseases cause massive economic losses by influencing the value of milk, meat, and other animal byproducts [35,36,37]. Among protozoan diseases, trypanosomiasis, or ‘Surra’, is a principally important and serious pathogenic protozoan disease of camels. There are several trypanosome species affecting livestock, and amongst them, *T. evansi* is the most prevalent in camels [6,7,38]. In Pakistan, Surra causes substantial sickness and death in camels and is ranked as the top priority among camel diseases.

Diagnosis of trypanosomiasis in animals is challenging due to parasitemic fluctuations, aparasitaemic intervals, and the difficulty in direct parasitedetection, especially in the sub-patent phase of infection [39]. The use of small blood smear samples requires an expert diagnostician but offers a straightforward way to directly identify hemoflagellates. It is a confirmatory method and a procedure used to detect protozoans in field conditions, although it has lower sensitivity compared to other molecular diagnostic techniques. Via comparison with microscopic blood smears, the PCR has demonstrated greater sensitivity, so it is widely endorsed for the diagnosis of trypanosomiasis [36].

Furthermore, PCR-based methods of detection have greater accuracy than conventional microscopic methods and are deliberated as the ‘gold standard’ for the diagnosis of parasitic infections [40]. For molecular detection of *T. evansi*, numerous sequences—e.g., the internal spacer transcribed region (ITS) [30], ribosomal DNA, kinetoplast DNA [41], and the variable surface glycoprotein (VSG) [42]—are considered reliable gene targets. In the present study, PCRs were performed using three sets of primers—ITS1CF/BR, Rotat 1.2 F/R, and pMUtec F/R—targeting variable genes with DNA extracted from camel blood samples. The PCR technique was found to be much more sensitive with all three sets of primers than the microscopic observation of blood smears. The PCR results revealed an overall infection rate of camel trypanosomiasis of 14.8%, higher than the microscopic findings (8.3%). These findings corroborate with those reported by the authors of [8], who determined overall infection of trypanosomiasis in Palestine to be 17% and 2.7% through PCR and microscopy, respectively. In another study, conducted in Somalia, the presence of Trypanosoma was detected in samples that were negative by microscopic examination [43]. Similar findings were derived from studies conducted in Nigeria [10] and Algeria [44]. The difference between the results of microscopy and molecular examination could be due to low levels of circulating parasites in the early phase of infection or chronic infection [45]. Moreover, the parasite is mostly visible in the blood smears in the febrile phase of disease. Therefore, due to the innate chronicity, trypanosomiasis often remains undetected by microscopy [46]. *T. evansi* was the single species documented as a major cause of camel trypanosomiasis in the investigation areas by ITS1CF/BR, Rotat 1.2, and pMUtec, in accordance with studies from Egypt [47] and Pakistan [9].

In the current study, microscopic examination of thin blood smears and DNA-based molecular assays such as PCR revealed the presence of *T. evansi* in camels (Camelus dromedarius). The overall rate of infection of *T. evansi* among the 400 suspected camels from ten districts of Punjab was 8.3% according to microscopic analysis of stained blood smears and 14.8% according to PCR. Both microscopic (5.3%) and PCR-based (11.1%) prevalence were lower in the camels of districts belonging to Northen Punjab, indicating that the camels of Southern Punjab were more at risk. The Giemsa-stained blood smear (GSBS)-based prevalence (8.3%) of *T. evansi* in the sampled camels was in accordance with the studies conducted in the Cholistan desert area [13,20,21]. These studies reported that the microscopic prevalence ranged between 9.7 and 5.4%. However, using GSBS, a higher prevalence of *T. evansi* was also found in Faisalabad [48], 19%, while a percentage of 32.5% was found in Attock [49], a percentage of 45.8% was found in Southern Punjab [24], and a percentage of 11.3% was found in Sindh province [50]. However, a lower rate of 0.7% was reported in the Cholistan desert [9], and a rate of 3.6% was found in the Khushab district [51]. The PCR-based prevalence (14.8%) found in this study was lower than past studies, with reports of 28.2% in Balochistan [52] and 39.3% in Cholistan desert [21]. Moreover, regarding the current investigation, the district-wise prevalence by RoTat 1.2 PCR for Bahawalpur (28.6%) and Bahawalnagar (22.7%) was higher when compared with previous studies [9,24]. The differences observed in prevalence could be attributed to the differences in the study designs, tests employed, sampling seasons, and sampling strategies. Furthermore, the selection of camels suspected of *T. evansi* infection could have inflated the prevalence estimates in this study. Moreover, the cross-sectional design did not allow us to investigate the seasonal variations caused by the fluctuations in vector density.

Globally, higher rates of *T. evansi* prevalence were recorded in various countries over different periods: 15.5% in Iran [53], 18% in UAE [54], 12.2% in Egypt [55], 20.9% and 23.4% in Egypt [56,57], 25.8% in Iran [58], 26.4% in Somaliland [46], and 31.5% in Nigeria [59]. Conversely, lower rates have been reported in various other countries: 5.3% in Nigeria [10], 2.7% in Palestine [8], 2.4% in Algeria [44], and 2.3% in Kenya [60]. Variations in infection rates may be attributed to several factors, such as climatic conditions, seasonal changes in sampling areas, animal population density, types of camel housing systems, differences in vector prevalence, availability of healthcare and diagnostic services, hygiene practices, or diversity in sample size [30].

The present research found that female camels were 2.1 times more likely to be positive, indicating that gender is a significant (*p* = 0.03) risk factor. This result agreed with the findings reported by other studies from Egypt [6], KSA [61], Iran [58], and Pakistan [9] but did not coincide with others reported by studies from Pakistan [52], Oman [62], and Ethiopia [63] that reported higher prevalence in male camels. A non-significant gender association with *T. evansi* prevalence was also reported in studies from Pakistan [13,41], Palestine [8], and Tunisia [38]. Variations in study design and diagnostic methodologies may account for the observed inconsistencies in research findings. The elevated prevalence in female camels identified in this study may be attributable to factors such as reproductive and lactational stress, hormonal influences, and differing management practices, all of which can affect exposure patterns, compromise immune function, and elevate disease risk [46].

Tick infestation (OR2.6: CI 1.37–4.79) was significantly associated with *T. evansi* prevalence (*p* = 0.003), but this may be confounded by other factors. There is no scientific evidence that ticks transmit *T. evansi*; rather, their presence may indicate environmental conditions favorable to biting flies, which also thrive in warm, humid areas [7]. Additionally, tick infestation can cause anemia and immunosuppression in camels, making them more susceptible to *T. evansi* infection [11].

Camels managed under arid sand-based housing systems demonstrated a 2.2-fold increased likelihood of testing positive for *T. evansi* (*p* = 0.01). Several potential confounding factors may account for this association, including the prevalence of nomadic pastoralism with extensive grazing [64], limited access to management and healthcare facilities [9], the use of communal water sources [65], and a higher density of vector populations in these environments. Additionally, animals with outdoor browsing habits may face elevated risk compared to those primarily browsing indoors due to greater exposure to vectors [66].

The serum biochemical analysis of infected (PCR-positive) camels showed a significant (*p* < 0.01) decrease in total protein, albumin, and globulin values, which is in accordance with studies conducted in camels [13,67], cattle [68], dogs [16], and rabbits [15]. However, these results were in contrast with those reported in camels [69] and buffaloes [70], showing increases and no significant changes in protein levels with *T. evansi* infection. The decrease in protein levels could be due to the severe hepatic degeneration and/or hypoxia in parasitic infection leading to hepatic necrosis and hypoalbuminemia [13,67]. With hyperglobulinemia in trypanosomiasis, hypoalbuminemia could be a compensatory practice to sustain osmolality [71].

Based on the findings of this study, it is recommended that a regionally coordinated control program be established to reduce the burden of *Trypanosoma evansi* infection in camels. Regular surveillance using PCR-based diagnostics should be adopted, particularly in high-risk areas of Southern Punjab, to enable early detection of both acute and carrier cases. Vector control must be prioritized through integrated measures such as insecticide application, environmental sanitation, and improved husbandry practices to limit mechanical transmission. Female camels, being more susceptible due to reproductive and physiological stress, should receive targeted monitoring, nutritional support, and prophylactic treatment. Finally, farmer education and community awareness programs should be implemented to promote recognition of clinical signs, adherence to treatment protocols, and adoption of preventive measures. Collectively, these actions will enhance camel health, productivity, and resilience against *T. evansi* infection while supporting the sustainability of camel-based livelihoods in Pakistan.

## 5. Conclusions

*T. evansi* (Surra) can significantly affect the economic stability of camel-rearing communities, who are dependent on these animals. Unlike earlier studies limited to one or a few districts in Punjab and other provinces of Pakistan, this is the first to combine multi-primer PCR detection, phylogenetic analysis, and risk factor evaluation of *T. evansi* across 10 major camel-rearing districts of Punjab, Pakistan. The results suggest that PCR diagnosis is a reliable tool for monitoring *T. evansi* infection in camels. Improving housing and health management for female camels is important, as they are at higher risk. However, the study’s cross-sectional design and sampling bias may reduce the generalizability of prevalence estimates. This study also did not consider seasonal changes in vector activity. Since *T. evansi* affects multiple species, future research should investigate mixed herds to clarify its epidemiology in Pakistan. Nationwide efforts to enhance diagnosis, treatment, and control are essential to reduce the disease’s economic impact.

## Figures and Tables

**Table 1:** Set of primers employed for molecular detection and sequencing.

Primer	Primer Sequence (5′ To 3′)	Expected Product (bp)	Reference
ITS1CF/BR	F: (CCGGAAGTTCACCGATATTG) R: (TTGCTGCGTTCTTCAACGAA)	480	[30]
pMUTec	F: (TGCAGACGACCTGACGTACT) R: (CTCCTAGAAGCTTCGGTGTCCT)	227	[31]
RoTat 1.2	F: (GCGGGGTGTTTAAAGCAATA) R: (ATTAGTGCTGCGTGTGTTCG)	205	[32]

**Table 2:** GenBank accession numbers of deposited sequences.

Accession No/ID	Parasite spp.
ON868415	*Trypanosoma evansi*
ON868416	*Trypanosoma evansi*
ON868417	*Trypanosoma evansi*
ON868418	*Trypanosoma evansi*
MZ209177	*Trypanosoma evansi*
MZ209178	*Trypanosoma evansi*

**Table 3:** District-wise infection rate of trypanosomiasis in camels by microscopy (n = 400).

District	Camel Population [18]	Tested	Microscopy		PCR	
			Positive	Prev. % (95% CI)	Positive	Prev. % (95% CI)
Jhang	1265	39	0	0 (0–9)	1	2.6 (0.1–13.5)
Faisalabad	687	15	1	6.7 (0.2–31.9)	2	13.3 (1.7–40.5)
Bhakkar	5310	90	7	7.8 (3.2–15.4)	14	15.6 (8.8–24.7)
Mianwali	1886	41	2	4.9 (0.6–16.5)	4	9.8 (2.7–23.1)
Khushab	3712	40	2	5 (0.6–16.9)	4	10 (2.8–23.7)
Rajanpur	7594	86	12	13.9 (7.4–23.1)	19	22.1 (13.9–32.3)
Muzaffargarh	1687	38	4	10.5 (2.9–24.8)	5	13.2 (4.4–28.1)
Bahawalpur	1078	14	2	14.3 (1.8–42.8)	4	28.6 (8.4–58.1)
Bahawalnagar	681	22	2	9.1 (1.1–29.2)	5	22.7 (7.8–45.4)
Layyah	3155	15	1	6.7 (0.2–31.9)	1	6.7 (0.2–31.9)
Total	27,055	400	33	8.3 (5.7–11.4)	59	14.8 (11.4–18.6)

Microscopic prevalence was not significantly different among the different sampled districts of Punjab (FET, *p* = 0.293). PCR-based prevalence was not significantly different among the different sampled districts of Punjab (FET, *p* = 0.097).

**Table 4:** Comparison of PCR and microscopy used to detect *T. evansi* in camels (n = 400).

PCR		Microscopy		Total
		Negative	Positive	
Negative	Count	316	25	341
	Expected Count	312.9	28.1	341.0
Positive	Count	51	8	59
	Expected Count	54.1	4.9	59.0
Total	Count	367	33	400
	Expected Count	367.0	33.0	400.0

**Table 5:** Agreement between using PCR and microscopy to detect *T. evansi* in camels (n = 400).

Comparison	Observed Agreement	SE	Kappa Value	95% CI of Kappa	Χ^2^ *p*-Value	Strength
PCR vs. MS	81.00%	0.057	0.076	−0.357, 0.188	0.108	Slight

**Table 6:** Results of risk factors associated with the trypanosomiasis in sampled camels (n = 400) from 10 districts of Punjab, Pakistan.

Variable	Category	Pos./Tested	Prev. % (95% CI)	Odds Ratio (95% CI)	*p*-Value
Provincial Zones	Northern and Central	25/225	11.1 (7.3–16)	Ref.	χ^2^ = 5.416 *p* = 0.020
	Southern	34/175	19.4 (13.8–26.1)	1.93 (1.10–3.38)	
Gender	Female	46/251	18.3 (13.7–23.7)	2.35 (1.22–4.51)	χ^2^ = 6.855 *p* = 0.009
	Male	13/149	8.7 (4.7–14.5)	Ref.	
Age Groups	<2 Y	10/87	11.5 (5.7–20.1)	Ref.	χ^2^ = 1.488 *p* = 0.475
	2–5 Y	21/149	14.1 (8.9–20.7)	1.26 (0.57–2.82)	
	>5 Y	28/164	17.1 (11.7–23.7)	1.59 (0.75–3.59)	
Tick Infestation	No	16/192	8.3 (4.8–13.2)	Ref.	χ^2^ = 12.090 *p* = 0.001
	Yes	43/208	20.7 (15.4–26.8)	2.87 (1.56–5.29)	
Wall Cracks	No	37/276	13.4 (9.6–18)	Ref.	χ^2^ = 1.279 *p* = 0.258
	Yes	22/124	17.7 (11.5–25.6)	1.39 (0.78–2.48)	
Contact with other Livestock	No	16/134	11.9 (7–18.7)	Ref.	χ^2^ = 1.265 *p* = 0.261
	Yes	43/266	16.2 (12–21.2)	1.42 (0.77–2.63)	
Physical appearance	Emaciated	51/307	16.6 (12.6–21.3)	2.12 (0.97–4.64)	χ^2^ = 3.642 *p* = 0.056
	Normal	8/93	8.6 (3.8–16.2)	Ref.	
Housing Management	Sand based	42/214	19.6 (14.5–25.6)	2.43 (1.33–4.43)	χ^2^ = 8.702 *p* = 0.003
	Soil based	17/186	9.1 (5.4–14.2)	Ref.	
Fly Control	No	42/296	14.2 (10.4–18.7)	Ref.	χ^2^ = 0.285 *p* = 0.594
	Yes	17/104	16.4 (9.8–24.9)	1.18 (0.64–2.18)	
Location of Feed and Water	Indoor	13/125	10.4 (5.7–17.1)	Ref.	χ^2^ = 2.736 *p* = 0.098
	Outdoor	46/275	16.7 (12.5–21.7)	1.73 (0.89–3.33)	
Purpose	Draught	33/190	17.4 (12.3–23.5)	1.49 (0.85–2.60)	χ^2^ = 1.973 *p* = 0.160
	Production	26/210	12.4 (8.2–17.6)	Ref.	
Herd Size	≤3	27/153	17.7 (12–24.6)	Ref.	χ^2^ = 2.127 *p* = 0.546
	4 to 6	11/99	11.1 (5.7–19)	0.58 (0.28–1.24)	
	7 to 10	12/87	13.8 (7.3–22.9)	0.75 (0.36–1.56)	
	>10	9/61	14.8 (7–26.2)	0.81 (0.36–1.84)	

**Table 7:** Results of binary logistic regression analysis indicating variables associated with *T. evansi* prevalence in sampled camels (n = 400) from 10 districts of Punjab, Pakistan.

Variable Name	Exposure Variable	Comparison	OR	95% CI	*p*-Value
Provincial Zones	Southern Punjab	Northern and Central Punjab	1.9	1.05–3.35	0.034
Gender	Female	Male	2.2	1.11–4.24	0.023
Tick Infestation	Yes	No	2.6	1.37–4.79	0.003
Housing Management	Sand Based	Soil Based	2.2	1.16–3.99	0.01

Model Fit: Nagelkerke R^2^ = 0.128; Hosmer–Lemeshow Test (χ^2^ = 7.038, *p* = 0.533).

**Table 8:** Comparison of serum biochemical parameters between *T. evansi*-positive (n = 59) and *T. evansi*-negative (n = 341) camels sampled from 10 districts of Punjab, Pakistan.

Parameters	Positive (n = 59)	Negative (n = 341)	*p*-Value
Total Protein (g/L)	55.1 ± 0.5	67.7 ± 0.8	<0.01
Albumin (g/L)	27.7 ± 0.4	36.5 ± 0.4	<0.01
Globulin (g/L)	25.7 ± 0.6	31.2 ± 0.8	<0.01
A\G Ratio	11.3 ± 0.4	11.8 ± 0.3	0.319

## Data Availability

The data presented in this study are available in NCBI Genbank at [https://www.ncbi.nlm.nih.gov/genbank/about/ (30 June 2025)] (reference number [MZ209177.1, MZ209178.1]).

## Supplementary Materials

The following supporting information can be downloaded at: https://www.mdpi.com/article/10.3390/vetsci12111055/s1. Figure S1: Original image of Figure 3; Figure S2: Original image of Figure 4; Figure S3: Original image of Figure 5.

## Abbreviations

The following abbreviations are used in this manuscript:

*T. evansi*	*Trypanosoma evansi*
FAT	Fisher’s exact test
PCR	Polymerase chain reaction
DNA	Deoxyribonucleic acid
BLAST	Basic Local Alignment Search Tool
CI	95% binomial exact confidence interval
PC	Positive control
NC	Negative control
CP	Control positive
CN	Control negative
MS	Microscopy
OR	Odds ratio
GSBS	Giemsa-stained blood smears
