# Supplementary material for: Molecular Identification and Phylogenetic Analysis of Trypanosoma evansi with Assessment of Associated Risk Factors in Camels (Camelus dromedarius) Across Ten Districts of Punjab, Pakistan
Source: Vet Sci. 2025 Nov 2;12(11):1055. doi: 10.3390/vetsci12111055 (PMC12656976; doi:10.3390/vetsci12111055)
Supplement: Supplementary file 1 [file vetsci-12-01055-s001.zip › vetsci-3856493-supplementary.pdf]

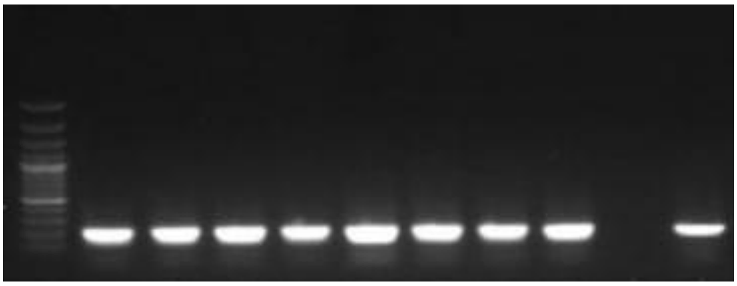

Figure S1 : Original image of Figure 3

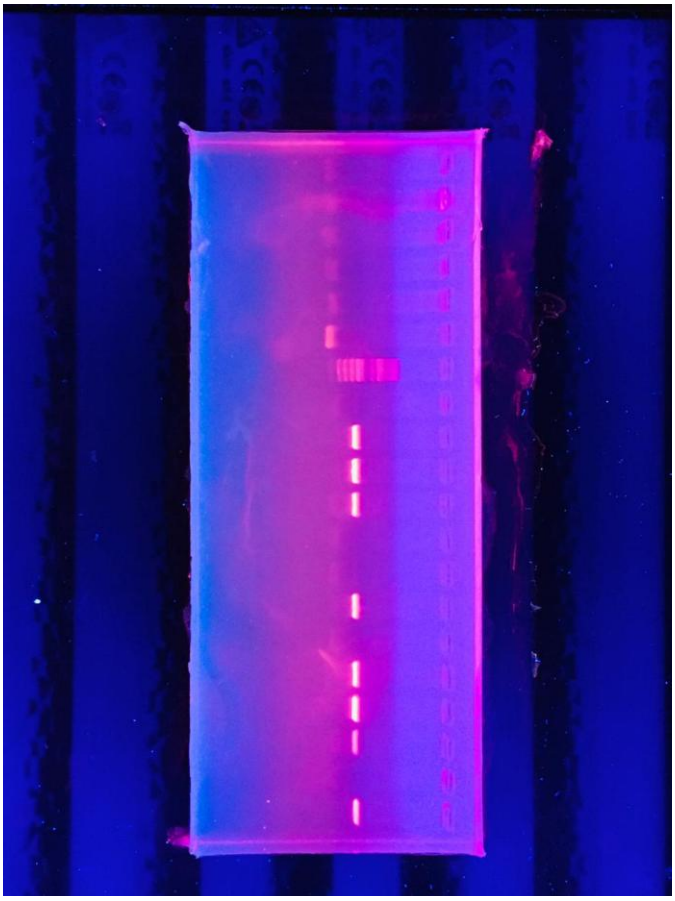

Figure S2 : Original image of Figure 4

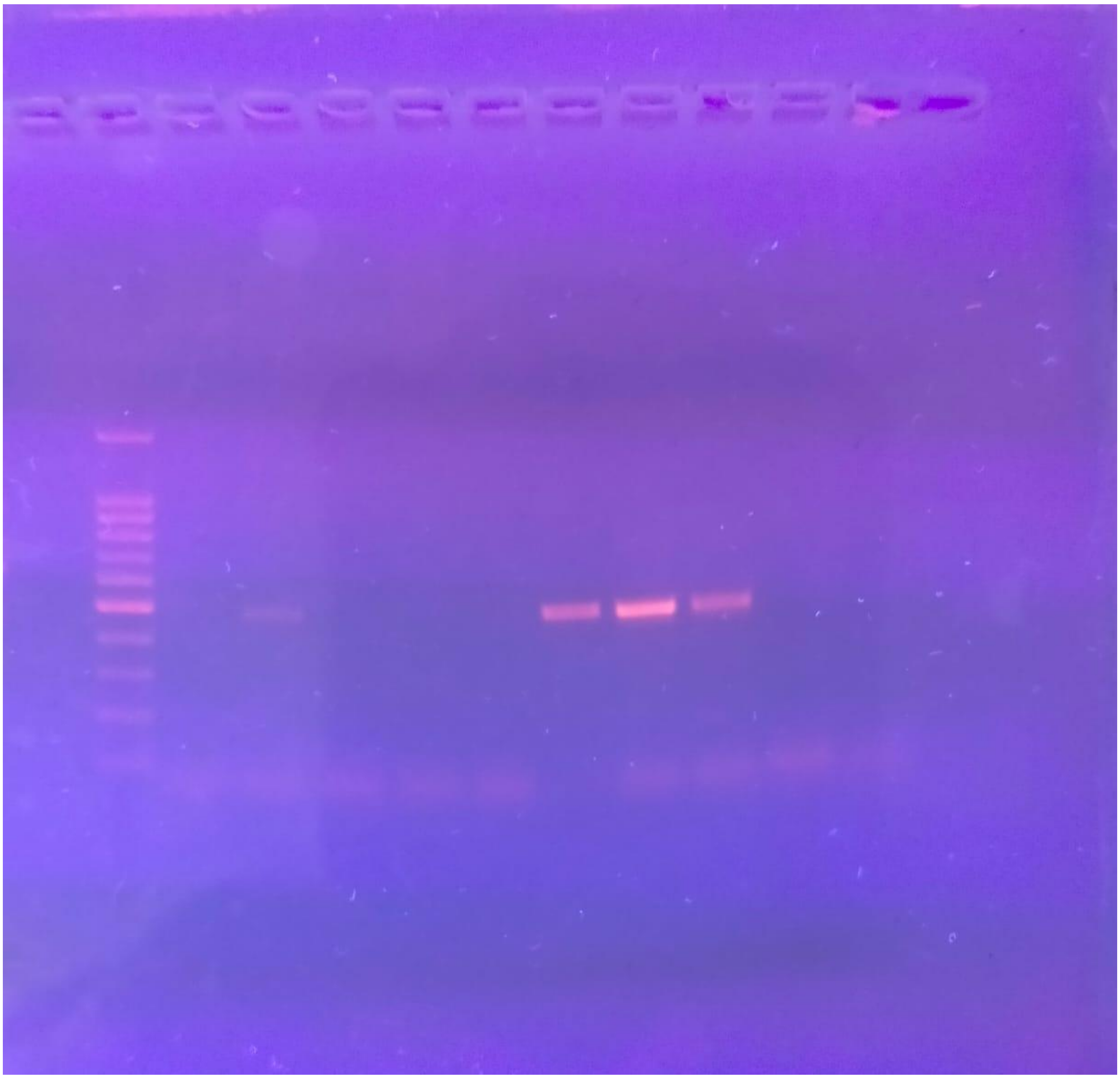

Figure S3 : Original image of Figure 5
